# Pleconaril and ribavirin in new-onset type 1 diabetes: a phase 2 randomized trial

**DOI:** 10.1038/s41591-023-02576-1

**Published:** 2023-10-04

**Authors:** Lars Krogvold, Ida Maria Mynarek, Erica Ponzi, Freja Barrett Mørk, Trine Witzner Hessel, Trine Roald, Nina Lindblom, Jacob Westman, Peter Barker, Heikki Hyöty, Johnny Ludvigsson, Kristian F. Hanssen, Jesper Johannesen, Knut Dahl-Jørgensen

**Affiliations:** 1 Division of Paediatric and Adolescent Medicine, Oslo University Hospital, Oslo, Norway; 2 Clinical Trial Unit, Oslo University Hospital, Oslo, Norway; 3 Steno Diabetes Center Copenhagen, Herlev University Hospital, Copenhagen, Denmark; 4 Department of Clinical Medicine, Faculty of Health and Medical Sciences, University of Copenhagen, Copenhagen, Denmark; 5 Apodemus AB, Stockholm, Sweden; 6 National Institute for Health and Care Research Cambridge Biomedical Research Centre, Core Biochemistry Assay Laboratory, Cambridge, UK; 7 Faculty of Medicine and Health Technology, Tampere University, Tampere, Finland; 8 Fimlab Laboratories, Tampere, Finland; 9 Linköping University, Linköping, Sweden; 10 Faculty of Medicine, University of Oslo, Oslo, Norway

**Keywords:** Randomized controlled trials, Virology, Innate immunity, Type 1 diabetes

## Abstract

Previous studies showed a low-grade enterovirus infection in the pancreatic islets of patients with newly diagnosed type 1 diabetes (T1D). In the Diabetes Virus Detection (DiViD) Intervention, a phase 2, placebo-controlled, randomized, parallel group, double-blind trial, 96 children and adolescents (aged 6–15 years) with new-onset T1D received antiviral treatment with pleconaril and ribavirin (*n* = 47) or placebo (*n* = 49) for 6 months, with the aim of preserving β cell function. The primary endpoint was the mean stimulated C-peptide area under the curve (AUC) 12 months after the initiation of treatment (less than 3 weeks after diagnosis) using a mixed linear model. The model used longitudinal log-transformed serum C-peptide AUCs at baseline, at 3 months, 6 months and 1 year. The primary endpoint was met with the serum C-peptide AUC being higher in the pleconaril and ribavirin treatment group compared to the placebo group at 12 months (average marginal effect = 0.057 in the linear mixed model; 95% confidence interval = 0.004–0.11, *P* = 0.037). The treatment was well tolerated. The results show that antiviral treatment may preserve residual insulin production in children and adolescent with new-onset T1D. This provides a rationale for further evaluating antiviral strategies in the prevention and treatment of T1D. European Union Drug Regulating Authorities Clinical Trials identifier: 2015-003350-41.

## Abstract (web summary)

Results from the DiViD Intervention, a phase 2 randomized, placebo-controlled trial, showed that antiviral treatment with pleconaril and ribavirin for 6 months resulted in higher endogenous insulin production in children and adolescents with new-onset type 1 diabetes.

## Main

Type 1 diabetes (T1D) is characterized by progressive loss of pancreatic β cell function that leads to lifelong dependence on insulin therapy^1^. Childhood-onset T1D is a severe disease not only because of disabling acute and late complications leading to shortened life expectancy, but also because of the heavy burden of a demanding therapy regimen on patients and their families^2^.

The disease is the result of a complex interplay between genetic predisposition, the immune system and environmental factors^3,4^. However, despite intensive biochemical, immunological, epidemiological and clinical research during the last 100 years, the etiology of T1D remains largely unknown. Some viruses can cause diabetes in animal models^5^. Viral infections can also contribute to the development of islet autoimmunity in humans^6^. Enteroviruses in particular have been implicated in the pathogenesis of T1D. Approximately 240 enterovirus types capable of infecting humans have been identified (https://www.picornaviridae.com/); enterovirus infections are common in childhood, ranging from an asymptomatic to a severe clinical presentation^7^. Meta-analyses showed a clinically significant association between enterovirus infections and the appearance of autoantibodies (T1D stages 1 and 2) and the onset of clinical diabetes (T1D stage 3) (refs. ^8–10^). Enterovirus capsid protein has also been detected in the β cells of patients with T1D^11^.

In the Diabetes Virus Detection study (DiViD), pancreatic tissue was collected from six adult patients with newly diagnosed T1D^12^. We demonstrated a low-grade enterovirus infection in the pancreatic islets in all patients and detected infection-competent enteroviruses in the pancreatic tissue from all cases^13–15^. Among known enteroviruses, only some may have diabetogenic properties. Coxsackie B enteroviruses have been associated with T1D in epidemiological studies^9^. Enteroviruses display tropism to the pancreatic islets because β cells strongly express the receptor used by these viruses^16^. The possible role of viral infection and the rationale for performing trials targeting enteroviruses were reviewed elsewhere^17^.

Pleconaril, developed against enteroviruses, clears viruses in β cell models of persistent enterovirus infection^18–20^. Pleconaril significantly reduced mortality due to severe enteroviral sepsis in neonates^21^. Ribavirin is a nucleoside analog with broad-spectrum antiviral activity against a variety of viruses, including enteroviruses^22,23^. An in vitro study showed that ribavirin may have an immunomodulatory effect, leading to enhanced interferon-γ (IFNγ) response and thus to a possible additive antiviral effect^24^.

Despite existing evidence for enteroviral infection initiating the autoimmune response and subsequent β cell destruction in genetically predisposed individuals, it is unclear if the main effect of the viruses is to initiate or drive the disease process, or both^25^. It is possible that during an acute enteroviral infection the virus spreads to the pancreas, infects β cells and then turns into a terminally deleted, replication-deficient form, which is characteristic of persisting enteroviral infections^26^. Based on these data, it is possible that eradication of this low-grade infection could improve the pancreas’s ability to secrete insulin after the onset of clinical disease. In this phase 2, randomized, double-blind, placebo-controlled clinical trial (DiViD Intervention), we investigated the effect of antiviral treatment on endogenous insulin production measured using C-peptide in children and adolescents with newly diagnosed T1D.

## Results

### Participants

**Fig. 1:**
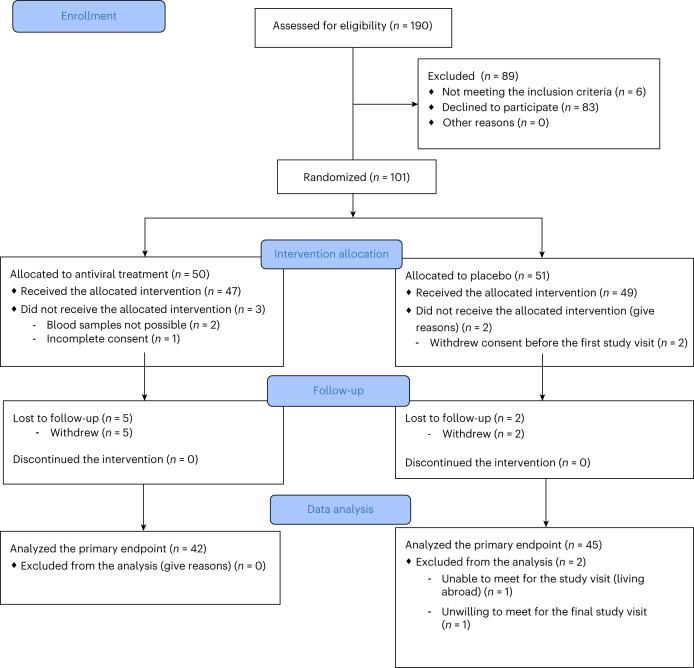
CONSORT diagram. CONSORT flow diagram for the DiViD interventional trial showing participant flow through each stage of the randomized controlled trial (enrollment, intervention allocation, follow-up and data analysis).

The trial included a screening period from the day of diagnosis until baseline (up to 3 weeks), a 26-week treatment period and a 26-week off-therapy follow-up period, and an ongoing extended follow-up of 2 additional years (to be reported). Out of 96 randomized participants, 47 (19 females and 28 males) were randomized to pleconaril and ribavirin and 49 (21 females and 28 males) to placebo. Participants were recruited between 20 August 2018 and 20 October 20. Details of the screening, randomization and follow-up of participants are provided in the CONSORT diagram (Fig. 1). Baseline anthropometric, clinical and metabolic characteristics are shown in Table 1.

**Table 1:** Characteristics of the trial participants at baseline

	All participants (*n* = 96)	Treatment group (*n* = 47)	Placebo group (*n* = 49)
Age, years (s.d.)	11.1 (2.4)	11.5 (2.3)	10.7 (2.5)
Females, *n* (%)^a^	40 (41.7)	19 (40.4)	21 (42.9)
Body mass index, kg m^−^^2^ (s.d.)	17.9 (2.7)	18.0 (2.7)	17.7 (2.7)
HbA1c at diagnosis, mmol mol^−1^ (s.d.)	105.8 (23.7)	110 (25.9)	102 (21.1)
HbA1c at diagnosis, % (s.d.)	11.8 (4.3)	12.2 (4.5)	11.5 (4.1)
Diabetic ketoacidosis at onset, %	12.5	10.6	14.3
Enterovirus present^b^	0	0	0
Anti-glutamic acid decarboxylase, *n* (%)	80 (83)	39 (83)	41 (84)
Islet antigen 2 antibodies, *n* (%)	74 (77)	35 (74)	39 (80)
Anti-insulin antibodies, *n* (%)	64 (67)	28 (60)	36 (73)
Anti-zinc transporter protein 8 antibodies, *n* (%)	77 (80)	37 (79)	40 (82)
0 antibodies, *n* (%)	1 (1)	1 (2.1)	0 (0)
1 antibody, *n* (%)	8 (8)	4 (8.5)	4 (8.2)
2 antibodies, *n* (%)	16 (17)	10(21.3)	6 (12.2)
3 antibodies, *n* (%)	29 (30)	13 (27.7)	16 (32.7)
4 antibodies, *n* (%)	42 (44)	19 (40.4)	23 (46.9)
Start of antiviral therapy, days from diagnosis (s.d.)	17.8 (3.2)	18.1 (3.2)	17.6 (3.2)

^a^Sex was reported by a pediatrician based on clinical examinations and interview with the participants.

^b^RT–PCR analyses of saliva, nasopharyngeal aspiration, nasal swab and feces.

### Primary endpoint

**Fig. 2:**
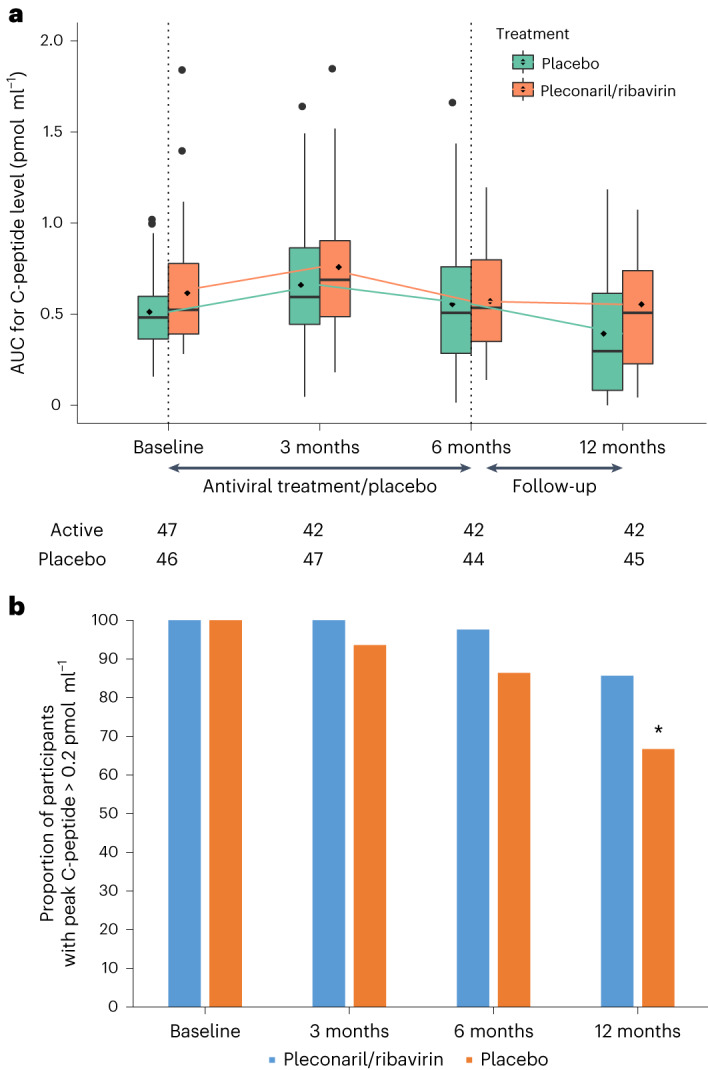
Residual insulin production. **a**, AUC for C-peptide levels. The box plots report the distribution of AUCs for the C-peptide levels. The numbers beneath the *x*axis reflect the number of participants in each group with the respective endpoint at each time point. The line inside the box represents the median; the box boundaries represent the 25th and 75th percentiles (the box is the interquartile range (IQR)); the whiskers represent the highest or lowest values within 1.5× the IQR; the points outside 1.5× the IQR represent the potential outliers, including the maximum and minimum values. The C-peptide levels after 12 months were higher in the pleconaril and ribavirin group compared with the placebo group (AME = 0.057, *P* = 0.037 from the linear mixed model). **b**, Peak stimulated C-peptide. The proportion of participants with peak serum C-peptide greater than 0.2 pmol ml^−1^ at different visits, divided according to antiviral treatment and placebo. *P = 0.04 in the logistic model; risk ratio = 1.29; two-sided 95% CI = 1.03–1.65.

The primary endpoint was endogenous insulin production at 12 months, as assessed by the 2-h serum C-peptide rea under the curve (AUC) during a mixed meal tolerance test (MMTT). Endogenous insulin production was measured at baseline, and then at 3, 6 and 12 months. The primary endpoint was analyzed using a linear mixed model for repeated measures. At 12 months, the serum C-peptide AUC was higher in the pleconaril and ribavirin treatment group compared to the placebo group (average marginal effect (AME) = 0.057 at 12 months in the linear mixed model; 95% confidence interval (CI) = 0.004–0.11, *P* = 0.037) (Fig. 2a).

**Extended Data Fig. 1:**
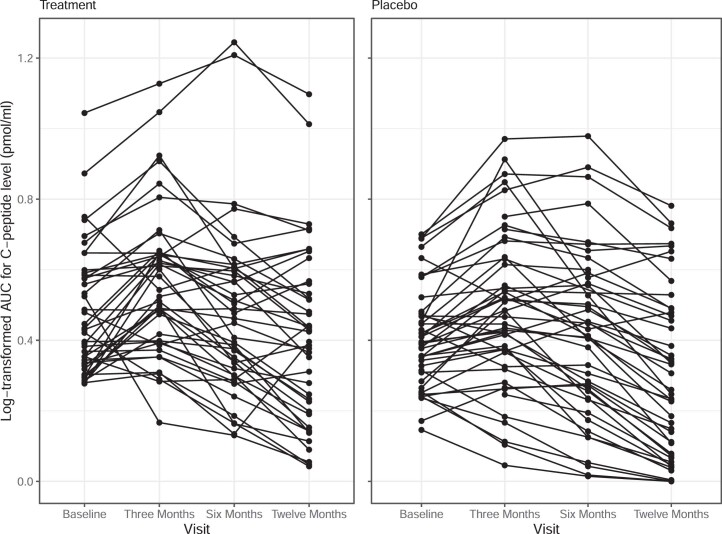
Individual trajectories of C-peptide AUC. Longitudinal plots with trajectories of log transformed C-peptide AUC, all participants, divided into treatment and placebo group. *N* = 91 (47 in placebo and 44 in active group).

At baseline, the mean (±s.d.) of the non-log-transformed 2-h C-peptide AUC was 0.62 (0.31) pmol ml^−1^ in the pleconaril and ribavirin group and 0.51 (0.22) pmol ml^−1^ in the placebo group (*P* = 0.07). At 12 months, the 2-h C-peptide AUC was 0.55 (0.42) pmol ml^−1^ in the pleconaril and ribavirin group and 0.39 (0.34) pmol ml^−1^ in the placebo group. During the 12-month study period, the relative decrease in C-peptide AUC was 11% in the pleconaril and ribavirin group and 24% in the placebo group. Figure 2a shows the AUC boxplots for the C-peptide levels in response to a 2-h MMTT. The observed imbalances in baseline values of the 2-h C-peptide AUC is accounted for by including the baseline values in the linear mixed model for the primary endpoint. Extended Data Fig. 1 shows the individual trajectories of the log-transformed C-peptide AUCs during the study period divided into pleconaril and ribavirin group and placebo group.

### Secondary endpoints

At baseline, all participants in both groups had a peak serum C-peptide secretion greater than 0.2 pmol ml^−1^ measured during MMTT. At 12 months, 36 of 42 participants (86%) in the pleconaril and ribavirin group and 30 of 45 participants (67%) in the placebo group secreted above this cutoff value (*P* = 0.04 in the logistic model, risk ratio = 1.29, 95% CI = 1.03–1.65) (Fig. 2b).

**Fig. 3:**
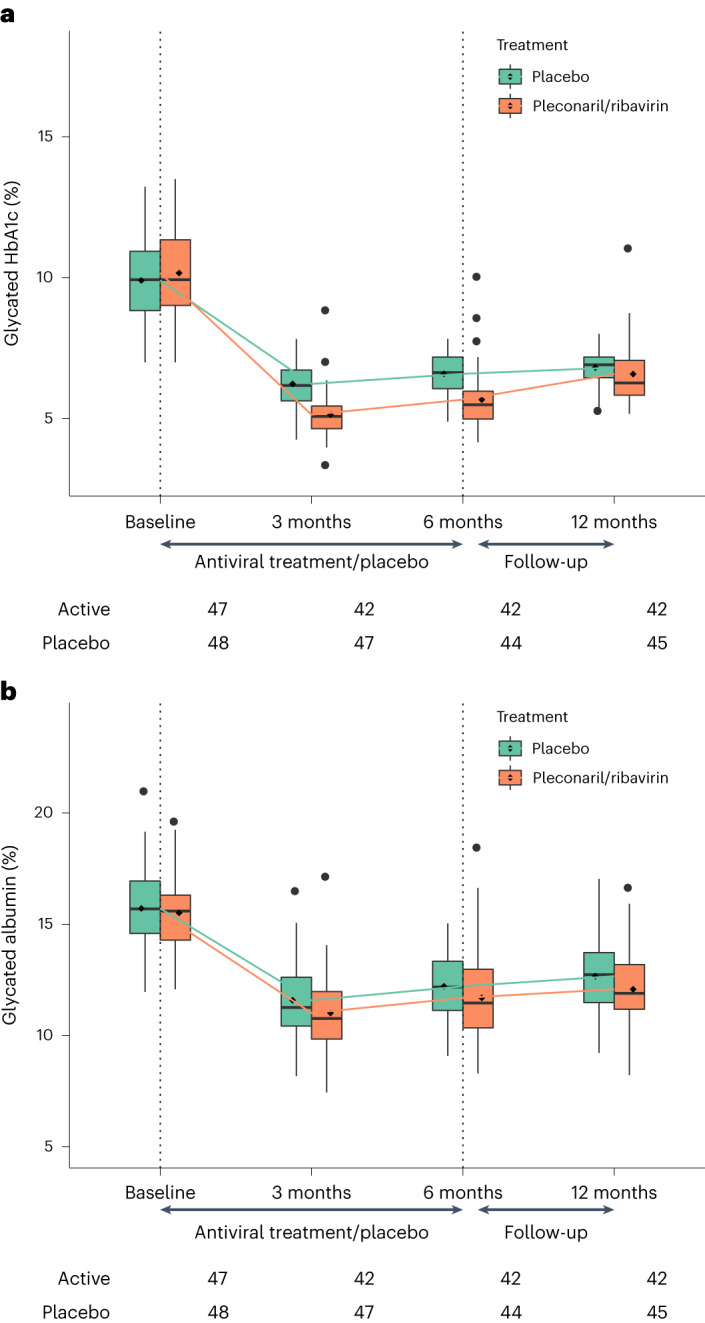
Metabolic control. **a**,**b**, The box plots show HbA1c (**a**) and glycated albumin (**b**), respectively. HbA1c was significantly lower in the pleconaril and ribavirin group compared to the placebo group at 3 and 6 months, but not at 12 months. Glycated albumin was not significantly different between groups at any time point. The numbers beneath the *x* axis reflect the number of participants in each group with the respective endpoint at each time point. The line inside the box represents the median; the box boundaries represent the 25th and 75th percentiles (the box is the IQR); the whiskers represent the highest or lowest values within 1.5× the IQR; the points outside 1.5× the IQR represent the potential outliers, including the maximum and minimum values.

HbA1c levels were similar in the pleconaril and ribavirin group and in the placebo group both at baseline (88 (18) versus 85 (16) mmol mol^−1^) and at 12 months (48 (12) versus 51 (9) mmol mol^−1^) (Table 2). At 3 and 6 months, HbA1c was lower in the pleconaril and ribavirin group compared to the placebo group (*P* < 0.0001; Table 2and Fig. 3a).

**Table 2:** Secondary endpoints

		Baseline	3 months	6 months	12 months
Percentage of participants with maximal stimulated serum C-peptide > 0.2 pmol ml^−1^	Treatment	100	100	97.6	85.7
	Placebo	100	93.6	86.4	66.7
HbA1c, mmol mol^−1^ (s.d.)	Treatment	87.6 (18.1)	32.7 (9.9)^*^	38.5 (12.5)^*^	48.4 (12.2)
	Placebo	84.7 (16.2)	44.6 (8.3)	48.5 (8.1)	50.9 (7.6)
HbA1c, % (s.d.)	Treatment	10.2 (3.8)	5.1 (3.1)^*^	5.7 (3.3)^*^	6.6 (3.3)
	Placebo	9.9 (3.6)	6.2 (2.9)	6.6 (2.9)	6.8 (2.8)
Glycated albumin, % (s.d.)	Treatment	15.5 (1.8)	11 (1.8)	11.7 (2.2)	12.1 (2)
	Placebo	15.7 (1.8)	11.6 (1.8)	12.2 (1.5)	12.7 (1.6)
IDAA1c, % (s.d.)	Treatment	13.2 (2.5)	7.2 (1.7)^*^	8.2 (2.2)^**^	9.9 (2.2)
	Placebo	12.8 (2.3)	8.2 (1.4)	8.9 (1.5)	9.9 (1.7)
Insulin dose, U kg^−1^ body weight per day (s.d.)	Treatment	0.8 (0.4)	0.8 (0.3)	1.1 (0.4)	1.3 (0.5)
	Placebo	0.7 (0.3)	0.8 (0.4)	0.9 (0.3)	1.1 (0.4)
Insulin pumps, *n* patients	Treatment	19	26	25	32
	Placebo	15	26	30	33

^*^P < 0.001, **P = 0.008. All other *P* values were not significant (*P* > 0.05).

Glycated albumin was similar in the pleconaril and ribavirin group and the placebo group at baseline and slightly, but not significantly, lower in the treatment group at 3, 6 and 12 months (Table 2 and Fig. 3b).

Insulin dose-adjusted HbA1c (IDAA1c) was similar in the pleconaril and ribavirin group and in the placebo group both at baseline (13.2% versus 12.8%) and at 12 months (9.9% versus 9.9%) (Table 2). At 3 and 6 months, IDAA1c was lower in the pleconaril and ribavirin group compared to the placebo group.

At baseline, the total daily insulin dose per body weight was similar in the pleconaril and ribavirin group and the placebo group (0.75 (0.37) U kg ^−1^day^−1^ versus 0.71 (0.30) U kg^−1^ day^−1^), and the insulin dose was not different between the two groups at any of the follow-up visits (Table 2).

Severe hypoglycemic events, defined as unconsciousness or seizures, were reported in two participants in the placebo group and none in the pleconaril and ribavirin group during the first year of the study (the follow-up part of the study is ongoing). Laboratory analysis of additional secondary endpoints (extended virus analysis and serum proinsulin:C-peptide ratio) will be performed at end of follow-up at 3 years, according to the protocol.

### Safety endpoints

A summary of the key safety events is shown in Table 3. Adverse events were recorded using the Common Terminology Criteria for Adverse Events v.5.0 from the National Institutes of Health. The incidence of adverse events during the first year was 93.6% (44 of 47 participants) in the pleconaril and ribavirin group and 95.9% (47 of 49 participants) in the placebo group. No serious adverse events were reported in either group. Hemolysis is a well-known side effect of ribavirin. Twenty-seven participants in the pleconaril and ribavirin group and 23 in the placebo group had markers of hemolysis in the laboratory analyses at some point across the first year. None had to reduce the dose of ribavirin because of anemia. There were no differences in the occurrence of infections, neither during treatment nor during 6 months after treatment. Two participants in the pleconaril and ribavirin group had a mild coronavirus disease 2019 (COVID-19) infection, 9 and 11 months after baseline (3 and 5 months after ending the treatment period), but none in the placebo group.

**Table 3:** Adverse events (safety analysis population) for the first year

	Pleconaril and ribavirin (*n* = 47)	Placebo (*n* = 49)
	*n* events (*n* patients)	
Any adverse event	188 (41)	137 (42)
Leading to discontinuation^a^	2	1
Possibly related to the trial agents^b^	130 (36)	81 (29)
Any serious adverse event	0 (0)	0 (0)
Death	0	0
Pregnancy	0	0
Any infection 0–6 months^c^	47 (30)	48 (32)
Any infection 0–12 months^c^	66 (35)	66 (36)
Serious infection	0	0
Serious hypoglycemia^d^	0	2 (2)
Hemolysis	80 (27)	55 (23)
Skin and subcutaneous disorders	4 (3)	7 (5)
Gastrointestinal symptoms	40 (21)	21 (13)
Psychological stress and anxiety	4 (1)	0

^a^One patient in the pleconaril and ribavirin group was withdrawn from the study on day 4 because of nausea; another patient in the same group withdrew on day 90 because of agitation and psychological stress. One patient in the placebo group withdrew from the study on day 36 because of abdominal pain.

^b^An adverse event related to the trial agent was defined as any event that was deemed by the trial investigators to be very likely, probably or possibly related to the trial drug or placebo or if the data regarding the relationship to the trial agent were missing.

^c^Recorded via questionnaire at each study visit.

^d^Defined as unconsciousness or seizures.

## Discussion

In this phase 2 trial involving children and adolescents with newly diagnosed T1D, combination therapy with the antiviral drugs pleconaril and ribavirin for 6 months resulted in a higher endogenous insulin production than placebo 12 months from baseline. The treatment was safe and tolerable.

Viruses contribute to the pathogenesis of T1D, both by damaging β cells or triggering the autoimmune response. Establishment of a low-grade persistent infection in β cells maintains inflammation, leading to the breakdown of tolerance to β cell antigens, β cell dysfunction and stress, each of which might then contribute to autoimmunity as part of a vicious circle^17^. The antiviral defense mechanisms of β cells are compromised compared to other islet cells^27,28^. Extensive peptide production by stressed β cells may increase the susceptibility to secreted malformed proteins, which may become antigenic and induce autoimmunity and T1D (stage 2) (refs. ^29^). This underlying disease mechanism of a low-grade, persistent viral infection may also apply to other autoimmune diseases, that is, cardiomyopathies, celiac disease, multiple sclerosis and Graves’ disease^30–35^.

Beneficial effects of antiviral treatment on β cell function have been observed in the present study at the onset of clinical stage 3 T1D, in a situation where disease progression had been ongoing for months or years. At this late stage of pathogenesis, patients might still have considerable numbers of β cells but they may not function normally^36,37^. The present study suggests that it could be possible to improve the function of the β cell mass with antiviral treatment. However, antiviral treatment took 12 months to demonstrate a difference in endogenous insulin secretion. This suggests that it may take a long time to experience the effects of antiviral treatment against a persistent, low-grade viral infection. In this study, a positive effect was observed in individuals younger than 15 years, who usually have profound islet inflammation with rapid loss of β cell function preceding diagnosis^36^. Therefore, it may be more effective to intervene with antivirals at an earlier stage of the disease, that is, stage 2, when there is more β cell function to preserve.

This trial was not designed to distinguish the individual effects of the two study drugs included in this treatment. The main reason for including ribavirin was to prevent the development of viral drug resistance. Ribavirin has a broader antiviral effect both on enteroviruses and several other viruses, and may also have immunomodulatory effects^22,23,38^. This potential immunomodulatory effect enhances IFNγ, leading to an additive antiviral effect in the context of an in vitro infection^24^.

The scale of the observed difference in C-peptide secretion between the pleconaril and ribavirin group and the placebo group after 12 months is comparable to the scale of improvement reported for anti-inflammatory medications and lately verapamil, which have been evaluated for newly diagnosed T1D in randomized control trials^39,40^. However, it is difficult to compare treatment effects across studies because of differences in study design and cohorts. The question around persistence of the observed difference at 12 months will be reported elsewhere, when all participants have been followed for 3 years.

The observed difference in C-peptide AUC at baseline was not significant (*P* = 0.07). Still, it could cause concern regarding the estimates of the treatment effect from the primary endpoint. For this reason, we adjusted for the baseline in the linear mixed model by including the baseline value among the repeated measurements.

The observed moderate effect of pleconaril and ribavirin may have clinical implications. In general, even a limited preservation of C-peptide levels is associated with improved metabolic control, reduced hypoglycemia and lower long-term microvascular complications^41^. However, a longer observation period is needed to clarify this issue in participants.

Lower HbA1c was observed in the pleconaril and ribavirin group during the antiviral treatment period (3 and 6 months from baseline) than in the placebo group; at the later time point, 6 months after the end of treatment, the difference in HbA1c between the groups was less pronounced. A known side effect of ribavirin is that it can induce hemolysis, leading to reduced lifespan of erythrocytes and lowering of HbA1c (ref. ^42^). The frequency and duration of hemolysis was highest in the pleconaril and ribavirin group (Table 3). Thus, we cannot exclude the possibility that this may, at least in part, explain the observed differences in HbA1c at 3 and 6 months. To compensate for the potential reduced lifespan of erythrocytes, we measured glycated albumin. No significant differences in glycated albumin between the groups were observed. Insulin doses changed as usual during the first year of insulin treatment, showing no significant differences between the treatment and placebo groups.

The trial has some limitations. First, the study included a small number of participants. The statistical power calculation for this trial was aimed at the primary endpoint and was not designed to estimate the power to detect secondary endpoints, which have greater between-person variation and laboratory analytical variation. Effects beyond those in the population studied, that is, centers, age, sex and potential role of COVID-19, could not be credibly examined. Second, the observed significant difference between the treatment and placebo group should be replicated using a higher number of participants of different ages and living in different countries, thus representing different microbiological environments. Third, information regarding continuous glucose monitoring was not available for all participants. Possible persisting low-grade enterovirus infection in the pancreas is difficult to detect from serum or samples taken from the periphery, even if the virus is detected in the pancreatic islets^15^. Because all trial participants were negative for enterovirus RNA using a sensitive PCR with reverse transcription (RT–PCR) in serum, stool and respiratory samples, we could not correlate the efficacy of treatment with the possible presence of such an infection.

In conclusion, this study shows that among children and adolescents with newly diagnosed T1D, the combination therapy of two antiviral drugs, pleconaril and ribavirin, resulted in higher residual endogenous insulin production than placebo. These results provide a rationale for future studies to evaluate the efficacy of antiviral drugs in the prevention and treatment of T1D.

## Methods

### Trial design

We conducted this phase 2, randomized, placebo-controlled, double-blind, parallel-group clinical trial at two sites: Oslo University Hospital, Norway and Steno Diabetes Center Copenhagen/Herlev University Hospital, Copenhagen, Denmark. Participants were children and adolescents aged 6–15 years with newly diagnosed T1D, randomly assigned in a 1:1 ratio to receive either a combination of pleconaril and ribavirin or placebo. We used block randomization, stratified according to site. The trial included a screening period from the day of diagnosis until baseline (up to 3 weeks), a 26-week treatment period and a 26-week off-therapy follow-up period, as well as an ongoing extended follow-up of two additional years (to be reported). The trial was conducted in accordance with the principles of the Declaration of Helsinki 2013, the International Conference on Harmonization Good Clinical Practice guidelines and applicable regulatory requirements. Approvals were obtained from the governmental and regional research ethics committees in Oslo and Copenhagen. Written informed consent was obtained from the participant’s caregiver and participants gave their verbal consent after receiving age-adjusted information. For the complete study protocol, see the Supplementary Information.

### Participants and randomization

Participants were aged between 6 and 15 years, had received a diagnosis of stage 3 T1D according to the American Diabetes Association criteria^8^. They were recruited from pediatric departments in southern Norway and in the Copenhagen Capital Region, Denmark between August 2018 and October 2020; they were able to undergo randomization within 3 weeks after the first insulin injection. Randomization was performed by statisticians at the Clinical Trial Support Unit in Oslo, having no contact with the trial personnel, and separate from the study centers. Masking success was ensured by the appointed good clinical practice (GCP) monitors.

The main inclusion criteria, which had to be fulfilled at screening before receiving the study agent, were: (1) diagnosed with T1D (International Statistical Classification of Diseases and Related Health Problems, 10th Revision code E10.9) with the first injection of insulin 3 weeks before inclusion; (2) willing and capable of taking the study drugs and meeting for tests and follow-up as described; (3) providing signed informed consent and expected to cooperate for the treatment and follow-up obtained and documented according to the International Council for Harmonisation of Technical Requirements for Pharmaceuticals for Human Use GCP, as well as national and local regulations; and (4) aged from 6.00 to 15.99 years at inclusion.

The main exclusion criteria were: (1) treatment with any oral or injected antidiabetic medication other than insulin; (2) a history of hemolytic anemia or significantly abnormal hematology results at screening; (3) history of severe cardiac disease in the previous 6 months; (4) impaired renal function; (5) participation in other clinical trials with a new chemical entity within the previous 3 months; (6) inability or unwillingness to comply with the provisions of the study protocol; (7) females who were lactating or pregnant; (8) males or females (after menarche) not willing to use highly effective contraception (progesterone-only hormonal anticonception with inhibition of ovulation or sexual abstinence) and barrier contraception (condoms), if sexually active during the treatment period and in the following 7 months; (9) presence of a serious disease or condition, which in the opinion of the investigators made the patient ineligible for the study.

### Treatment

Pleconaril and ribavirin were administered as oral solutions (to enable weight-based dosing) at home as separate mixtures for 26 weeks. Combination treatment was chosen to increase and broaden the antiviral effect and to reduce the risk of emergence of drug-resistant virus variants. A 6-month treatment was chosen based on clinical experience from treating other chronic viral infections. In cell models, pleconaril eradicated persistent enterovirus infection in 5–6 weeks^20^. The pleconaril dose was 5 mg kg^−1^ twice daily. The maximum daily dose was 600 mg. The ribavirin dose was 7.5 mg kg^−1^ twice daily. The maximum daily dose was 1,000 mg if body weight was less than 75 kg and 1,200 mg if body weight was more than 75 kg. Study drugs were delivered by Apodemus AB. Investigators and participants were blinded to the study drugs and placebo. Drugs or placebo were distributed to patients at the study visits to be administered at home and taken twice daily together with food. For details, see the Supplementary Information. Compliance was assessed by interviewing participants and care providers, and by measuring the returned drug bottles at each study visit.

### Endpoints

The primary endpoint was endogenous insulin production, as assessed according to the 2-h serum C-peptide AUC during an MMTT at 12 months. The AUC was calculated at each visit using the trapezoidal rule on five measurements collected during the 2-h test (at 0, 15, 30, 60 and 90 min, respectively). Secondary endpoints included a preserved peak C-peptide level greater than 0.2 pmol ml^−1^ during MMTT, insulin dosage, HbA1c, glycated albumin and severe hypoglycemic events.

### Laboratory methods

All laboratory analyses were performed with the same methods throughout the trial for all participants. All sampling and biobanking followed the INNODIA Master Protocol and Standard of Procedures^43^. MMTT was performed in the morning at the hospital in the fasting state with blood sampling at baseline and 15, 30, 60, 90 and 120 min after ingestion of a standardized liquid meal (Ensure Plus or equivalent, 6 ml kg^−1^ bodyweight, maximum 360 ml)^44^. For participants with missing time points during the MMTT, a weighted average of the available time points was used.

HbA1c was analyzed using international standard methods (coefficient of variantion (CV) 2%). Glycated albumin (%) was determined with liquid chromatography–tandem mass spectrometry (CV 2–6%) (ref. ^45^). Safety-related laboratory tests were performed using standard hospital assays.

Enterovirus RT–PCR was performed using nasal swab, nasopharyngeal aspirates, saliva, stool and serum samples as described previously^46^.

### Statistical analysis

The sample size calculation is based on the efficacy continuous variable ‘2-h AUC C-peptide’. Because this variable is strongly skewed to the right, a logarithmic transformation (log(*x* + 1)) is used. By using X data from large studies^47–49^ and claiming an 85% test power to detect a 50% absolute improvement with a 5% significance in the treatment group compared to the placebo group (mean = 0.179, s.d. = 0.172) after 12 months^45^. Eighty-six patients must be included in each group. This sample size calculation (*n* = 172) was based on an independent samples *t*-test when comparing 2-h AUC C-peptide values after 1 year. A recent publication showed that when using analysis of covariance, adjusting for baseline ‘AUC C-peptide’ and age, instead of a *t*-test, sample size may be reduced by 50% (ref. ^47^). As we expected a dropout rate of 10%, we decided to include 96 (86 + 10) patients in the study.

Both efficacy and safety analyses included all participants who had received at least one dose of pleconaril and ribavirin or placebo.

The primary null hypothesis was evaluated in the full analysis set (FAS), defined as all participants fulfilling the entry criteria who were randomly assigned to a treatment group. Sensitivity analysis was performed in the per protocol analysis set, which included all participants in the FAS who met the study eligibility criteria, with no major protocol deviations affecting treatment efficacy, and who complied with the prescribed treatment. Safety data were analyzed for all participants having taken the study medication. Individuals who withdrew from the study were included in the safety analysis. The primary endpoint was analyzed using a linear mixed model for repeated measures that was fitted on the log-transformed C-peptide AUC data. The model used the longitudinal log-transformed serum C-peptide AUCs at baseline, 3 months, 6 months and 1 year as the response variable and included categorical effects of site, treatment, time and treatment-by-time interaction, as well as a random effect for the patient ID to account for repeated measurements. The linear mixed model included all repeated measurements of C-peptide AUCs, which were available for 89 participants (42 in the pleconaril and ribavirin group and 47 in the placebo group, of which 87 had the 12-month measurement and the remaining 2 only had the previous measurements). The treatment effect was defined as the AME; the primary estimate of treatment effect was obtained as the AME at 12 months^50^.

The detailed statistical analysis plan (SAP) was produced before the final database lock, before extracting files from the INNODIA clinical database and before unblinding of treatment allocation. The SAP was signed by the authorized chief statistician of Oslo University Hospitals.

We fitted a linear mixed model on the mean residual insulin secretion, using the values collected at baseline, 3 months, 6 months and 12 months as a longitudinal outcome variable, and including a random effect for patient ID to account for dependency among measurements taken for the same individual. We included categorical effects for the study site, treatment, time and an interaction term between treatment and time. Because baseline values were collected before randomization, the treatment effect was set to 0 at baseline. Including the baseline value as one of the repeated measurements and forcing the treatment effect to be null at baseline is not only equivalent to adjusting for baseline, but is also the preferred alternative to analyze longitudinal endpoints^51,52^.

The strength of the model we used lies in the fact that we also included the measurements taken at 3 and 6 months, which provides additional power for the analysis. Using exclusively the change from baseline to 12 months would have resulted in less power. The present study had 96 participants; only 87 had records at 12 months. Eighty-nine patients had records at 3 or 6 months; such information can be used to strengthen the conclusion of the analyses. The measurements at 3 and 6 months were listed in the protocol as secondary endpoints; we analyzed them simultaneously in the proposed model. To ensure that we did not incur issues of multiplicity, we defined the primary treatment effect as the treatment effect at 12 months, which is consistent with the protocol. Including the interaction between time and treatment in the model allows us to do so by defining the treatment effect at 12 months as the AME at 12 months.

The use of multiple measurements reduces the uncertainty and increases the power of the findings, thus resulting in a statistically significant (*P* = 0.037) effect of the treatment at 12 months, while simultaneously preserving the adjustment for baseline and being equivalent to analyzing the change from baseline values.

To investigate the robustness of the results, we also ran a Wilcoxon *t*-test on the change from baseline to 12 months without including other repeated measurements. Although such a test results in a non-significant (*P* = 0.19) effect, it confirms the same trend toward efficacy and a similar estimate of the treatment effect as found by the linear mixed model. The loss of significance is most probably due to the reduced power when using only the 12-month measurements. The increased power and the ability to adjust for baseline values are at the basis of our model choice for the primary analysis. Nevertheless, this, and the other sensitivity analyses we conducted, all pointed in the same direction, although not all of them showed a significant effect. This pattern was consistent with the level of significance observed in the primary analysis (0.04) and confirmed the need for a larger follow-up study to confirm these findings.

The secondary endpoints of mean insulin dosage per body weight, HbA1c, glycated albumin and insulin dose-adjusted HbA1c, were analyzed using a linear mixed model for repeated measures with the same structure and covariates as for the primary endpoint.

The dichotomous secondary endpoint corresponding to the proportion of patients with peak C-peptide above 0.2 pmol ml^−1^ at 12 months was analyzed with a logistic model including site and categorical treatment as covariates. Missing data for all continuous missing outcomes were assumed to be missing at random and were imputed implicitly using a mixed model for repeated measures.

Results are reported as the mean (s.d.) for continuous outcomes and *n* (%) for binary outcomes. All reported *P* values are two-sided.

A safety and data monitoring committee was established before starting participant recruitment (committees members are listed in the ‘Acknowledgements’).

The trial was monitored closely with regard to safety. The data monitoring committee assessed the safety data and adverse events through the course of the trial. Efficacy data (primary endpoint) were not analyzed before unblinding. Therefore, no interim analysis was performed on the primary endpoint and no adaptive design or error rate control was needed.

### Reporting summary

Further information on research design is available in the Nature Portfolio Reporting Summary linked to this article.

## Online content

Any methods, additional references, Nature Portfolio reporting summaries, source data, extended data, supplementary information, acknowledgements, peer review information; details of author contributions and competing interests; and statements of data and code availability are available at 10.1038/s41591-023-02576-1.

### Supplementary information

Supplementary Information Supplementary treatment description, Tables 1 and 2 and legend to Extended Data Fig. 1.

Reporting Summary

## Data Availability

Data collected for the study and presented herein will be made available to others when the end-of-trial reports, after 3 years of follow-up, have been published. Anonymized participant data can be obtained upon reasonable request from the corresponding author. Proposals will be reviewed on the basis of scientific merit, ethical review, available resources and regulatory requirements. After approval of a proposal, anonymized data will be made available for reuse. The corresponding author has the right to review and comment on any draft papers based on these data before publication. Availability will follow General Data Protection Regulations. Data will be organized in a data dictionary and participant data will be de-identified. Related study documents, including the informed consent forms (in Norwegian) will also be available. The study protocols and the SAP are found in the Supplementary Information. All data requests should be sent to the corresponding author (knut.dahl-jorgensen@medisin.uio.no).

## Code availability

All the code used for the descriptive tables and the analysis of the primary and safety endpoints is publicly available at https://github.com/ericaponzi/DiViDInt-R-code.
