# Supplementary material for: Pleconaril and ribavirin in new-onset type 1 diabetes: a phase 2 randomized trial
Source: Nat Med. 2023 Oct 4;29(11):2902–8. doi: 10.1038/s41591-023-02576-1 (PMC10667091; doi:10.1038/s41591-023-02576-1)
Supplement: Supplementary file 1 — Supplementary treatment description, Tables 1 and 2 and legend to Extended Data Fig. 1. [file 41591_2023_2576_MOESM1_ESM.pdf]

nature medicine

Article https://doi.org/10.1038/s41591-023-02576-1

Pleconaril and ribavirin in new-onset type 1 diabetes: a phase 2 randomized trial

In the format provided by the authors and unedited

Supplement

Contents Page

Treatment 2

Supplementary table 1: The composition of the pleconaril placebo solution 2

Supplementary table 2: The composition of the ribavirin placebo solution 3

Reference list 3

Legend to Supplementary Figure 1. Individual trajectories of C-peptide AUC 3

Treatment

The drug product pleconaril was administrated as a 40 mg/ml solution with the triglyceride Crodamol (MCT-oil) as the only excipients. Similar formulations have been used by Viropharma in clinical trials with Pleconaril [1,2]. The drug product Ribavirin was administrated as a 40 mg/ml solution. The product is more complex compare to the pleconaril product formulation and contain the following excipients, sodium citrate, citric acid, sodium benzoate, glycerol, sorbitol, propylene glycol, water. The selected formulation is almost identical with the formulation used in the approved ribavirin product Rebetol (Merck Sharp & Dome). The only difference is that the current formulation lack glucose but contains higher amount of sorbitol and glycerol.

The patients were all hospitalized at diagnosis, and started insulin treatment according to ISPAD guidelines [3], by either insulin pumps or basal-bolus regimens with insulin pens, guided by finger prick home blood glucose monitoring or glucose sensors. Aim for metabolic control was HbA1c less than 53 mmol/mol (7.0 %).

Tables

Supplementary table 1

The composition of the pleconaril placebo solution

Component	*Content*	*Function*	*Reference*
Crodamol (MCT-oil)	100%	Oil solution	Ph. Eur.

Supplementary table 2

The composition of the ribavirin placebo solution

Component	*Content*	*Function*	*Reference*
Sodium Citrate dehydrate	5.27	Buffer	Ph. Eur.
Citric acid anhydrous	2.15	Buffer	Ph. Eur.
Sodium benzoate	1.0	Preservative	Ph. Eur.
Glycerol Anhydrous	250.0	Solvent	Ph. Eur.
Sorbitol Solution /70/70	300.0	Solvent/swee tener	Ph. Eur.
Propylene glycol	100.0	Solvent	Ph. Eur.
Tutti Frutti	0.25	Taste	Internal specification
Purified water	Qs 1L	Solvent	Ph. Eur

Reference list

1. Kearns, G.L., *et al.* Single-dose pharmacokinetics of a pleconaril (VP63843) oral solution in children and adolescents. Pediatric Pharmacology Research Unit Network. *Antimicrob. Agents Chemother* **43**, 634-638 (1999).
2. Hayden, F.G., *et al.* Oral pleconaril treatment of picornavirus-associated viral respiratory illness in adults: efficacy and tolerability in phase II clinical trials. *Antivir. Ther* **7**, 53-65 (2002).
3. Danne, T., *et al.* ISPAD Clinical Practice Consensus Guidelines 2018: Insulin treatment in children and adolescents with diabetes. *Pediatr. Diabetes. 2018. Oct*, 115-135 (2018).

Supplementary figure 1

Legend to Figure 1 Extended Data

Longitudinal plots with trajectories of log transformed C-peptide AUC, all participants, divided into treatment and placebo group. N= 91 (47 in placebo and 44 in active group)
