# Supplementary material for: Pleconaril and ribavirin in new-onset type 1 diabetes: a phase 2 randomized trial
Source: Nat Med. 2023 Oct 4;29(11):2902–8. doi: 10.1038/s41591-023-02576-1 (PMC10667091; doi:10.1038/s41591-023-02576-1)
Supplement: Supplementary file 2 — Reporting Summary [file 41591_2023_2576_MOESM2_ESM.pdf]

Corresponding author(s): Knut Dahl-Jorgensen

Last updated by author(s): 2023/08/30

Reporting Summary

Nature Portfolio wishes to improve the reproducibility of the work that we publish. This form provides structure for consistency and transparency in reporting. For further information on Nature Portfolio policies, see our Editorial Policies and the Editorial Policy Checklist.

Statistics

For all statistical analyses, confirm that the following items are present in the figure legend, table legend, main text, or Methods section.

n/a	Confirmed	
☐	☒	The exact sample size (*n*) for each experimental group/condition, given as a discrete number and unit of measurement
☐	☒	A statement on whether measurements were taken from distinct samples or whether the same sample was measured repeatedly
☐	☒	The statistical test(s) used AND whether they are one- or two-sided<br>*Only common tests should be described solely by name; describe more complex techniques in the Methods section.*
☐	☒	A description of all covariates tested
☐	☒	A description of any assumptions or corrections, such as tests of normality and adjustment for multiple comparisons
☐	☒	A full description of the statistical parameters including central tendency (e.g. means) or other basic estimates (e.g. regression coefficient) AND variation (e.g. standard deviation) or associated estimates of uncertainty (e.g. confidence intervals)
☐	☒	For null hypothesis testing, the test statistic (e.g. *F*, *t*, *r*) with confidence intervals, effect sizes, degrees of freedom and *P* value noted<br>*Give P values as exact values whenever suitable.*
☒	☐	For Bayesian analysis, information on the choice of priors and Markov chain Monte Carlo settings
☐	☒	For hierarchical and complex designs, identification of the appropriate level for tests and full reporting of outcomes
☒	☐	Estimates of effect sizes (e.g. Cohen's *d*, Pearson's *r*), indicating how they were calculated

Our web collection on statistics for biologists contains articles on many of the points above.

Software and code

Policy information about availability of computer code

Data collection	No commercial, open source software were used in Innodia Consortium database solution. www.innodia.eu.
Data analysis	Software: Data analysis was R version 3.6.1 (RStudio version: RStudio 2022.07.2+576). All the code used for the descriptive tables, the analysis of the primary endpoint, as well as the safety endpoints is publicly available at https://github.com/ericaponzi/DiViDInt-R-code.

For manuscripts utilizing custom algorithms or software that are central to the research but not yet described in published literature, software must be made available to editors and reviewers. We strongly encourage code deposition in a community repository (e.g. GitHub). See the Nature Portfolio guidelines for submitting code & software for further information.

Data

Policy information about availability of data

All manuscripts must include a data availability statement. This statement should provide the following information, where applicable:

- Accession codes, unique identifiers, or web links for publicly available datasets
- A description of any restrictions on data availability
- For clinical datasets or third party data, please ensure that the statement adheres to our policy

Data collected for the study and presented herein will be made available to others when the end of trial reports after three years of follow-up have been published. Anonymized participant data can be made available upon requests directed to the corresponding author. Proposals will be reviewed on the basis of scientific merit, ethical review, available resources and regulatory requirements. After approval of a proposal, anonymized data will be made available for reuse. The corresponding

author will have the right to review and comment on any draft papers based on these data before publication. The availability will follow General Data Protection Regulations. Data will be organized in a data dictionary, and participant data will be de-identified. Related study documents, including the informed consent forms (in Norwegian) will also be available. Study protocols, and statistical analysis plan is enclosed in Supplementary information. All data requests should be sent by email to the corresponding author (knut.dahl-jorgensen@medisin.uio.no).Data collected for the study and presented herein will be made available to others when the end of trial reports after three years of follow-up have been published. Anonymized participant data can be made available upon requests directed to the corresponding author. Proposals will be reviewed on the basis of scientific merit, ethical review, available resources and regulatory requirements. After approval of a proposal, anonymized data will be made available for reuse. The corresponding author will have the right to review and comment on any draft papers based on these data before publication. The availability will follow General Data Protection Regulations. Data will be organized in a data dictionary, and participant data will be de-identified. Related study documents, including the informed consent forms (in Norwegian) will also be available. Study protocols, and statistical analysis plan is enclosed in Supplementary information. All data requests should be sent by email to the corresponding author (knut.dahl-jorgensen@medisin.uio.no).

Human research participants

Policy information about studies involving human research participants and Sex and Gender in Research.

Reporting on sex and gender	The participants were recruited independent of sex, and the distribution was as usual in new onset type 1 diabetes. 42% were females. There were no sex differences in key clinical data at inclusion, and the treatment group and placebo group were well balanced on sex. Sex based analyses were made, but showed no significant differences in outcome parameters.
Population characteristics	The participants were between six and 15 years of age, had received a diagnosis of stage 3 type 1 diabetes according to American Diabetes Association (ADA) criteria, and were able to undergo randomization within three weeks after first insulin injection.
Recruitment	Participants were recruited from pediatric departments in Southern Norway and in Copenhagen Capital Region, Denmark between August 2018 and October 2020. All patients admitted with new onset type 1 diabetes were informed about the study, and asked for participation. No biases were intended or observed in respect of volunteering for the study.
Ethics oversight	Approvals were obtained from Regional Committees for Medical and Health Research Ethics in Oslo and Copenhagen. Written informed consent from the participant's caregiver were obtained and the participants gave their verbal consent after receiving age-adjusted information.

Note that full information on the approval of the study protocol must also be provided in the manuscript.

Field-specific reporting

Please select the one below that is the best fit for your research. If you are not sure, read the appropriate sections before making your selection.

☒ Life sciences ☐ Behavioural & social sciences ☐ Ecological, evolutionary & environmental sciences

For a reference copy of the document with all sections, see nature.com/documents/nr-reporting-summary-flat.pdf

Life sciences study design

All studies must disclose on these points even when the disclosure is negative.

Sample size	The sample-size calculation was based on the number of participants who would need to be enrolled to detect a significant difference between the treatment and placebo groups with respect to the primary end point. Owing to skewed C-peptide AUC data, a logarithmic transformation (log [AUC+1]) was performed to normalize the distribution. We calculated that a sample size of 86 participants and a randomization ratio of 1:1 between the pleconaril/rigroup would provide the trial 85% power to detect a 50% treatment difference at an alpha level of 0.05 (two-sided) based on ANOVA. To allow for a dropout rate of 10%, 96 participants were included in the study.
Data exclusions	No data were excluded.
Replication	The clinical data can not be replicated.
Randomization	The participants were children and adolescents aged 6-15 years with newly diagnosed type 1 diabetes randomly assigned in a 1:1 ratio to receive either a combination of pleconaril and ribavirin or placebo. We used block randomization, stratified by site.
Blinding	Randomization was performed by computer in the Clinical Trial Support Unit in Oslo, by statisticians having no contact to the trial personnel, and separate from the study centers. Success of masking were assured by the appointed GCP Monitors. Participants were given an unique study participant ID number used in the clinical data bank. Study drugs and placebo were blinded to investigators and participants.

Reporting for specific materials, systems and methods

We require information from authors about some types of materials, experimental systems and methods used in many studies. Here, indicate whether each material, system or method listed is relevant to your study. If you are not sure if a list item applies to your research, read the appropriate section before selecting a response.

Materials & experimental systems

n/a	Involved in the study
☒	☐ Antibodies
☒	☐ Eukaryotic cell lines
☒	☐ Palaeontology and archaeology
☒	☐ Animals and other organisms
☐	☒ Clinical data
☒	☐ Dual use research of concern

Methods

n/a	Involved in the study
☒	☐ ChIP-seq
☒	☐ Flow cytometry
☒	☐ MRI-based neuroimaging

Clinical data

Policy information about clinical studies

All manuscripts should comply with the ICMJE guidelines for publication of clinical research and a completed CONSORT checklist must be included with all submissions.

Clinical trial registration

EudraCT (number2015-003350-41, registered starting date 2017-03-28)

Study protocol

The full study protocol is enclosed in the Supplements

Data collection

The participants were between six and 15 years of age, had received a diagnosis of stage 3 type 1 diabetes according to American Diabetes Association (ADA) criteria. They were recruited from pediatric departments in Southern Norway and in Copenhagen Capital Region, Denmark between August 2018 and October 2020, and were able to undergo randomization within three weeks after first insulin injection. Randomization was performed by computer in the Clinical Trial Support Unit in Oslo, by statisticians having no contact to the trial personnel, and separate from the study centers. Success of masking were assured by the appointed GCP Monitors. Clinical data were collected according to protocol by study nurses and the investigators at each study visit in electronic Case record forms and stored in the INNODIA clinical database in Copenhagen. Materials/samples were collected according to the INNODIA Standard of procedures and analyzed in approved laboratories and results entered directly into the database.

Outcomes

Primary and secondary endpoints are described in detail in the Method section in the manuscript.
